# Skeletal Muscle Mass in Women With Breast Cancer: Evaluation of Agreement Between Different Methods

**DOI:** 10.1155/jnme/6646504

**Published:** 2026-08-24

**Authors:** Julia Abdala Nogueira Souza, Deborah Minto dos Santos, Luisa Barcellos Leite da Silva, Maria Rita Pereira da Silva Garcia, Ana Beatriz Rechinelli, Janine Martins Machado, Ben-Hur Albergaria, Rayne de Almeida Marques Bernabé, José Luiz Marques-Rocha, Valdete Regina Guandalini

**Affiliations:** ^1^ Department of Nutrition, Health Science Center, Federal University of Espírito Santo, Vitória, Espírito Santo, Brazil, ufes.br; ^2^ Postgraduate Program in Nutrition and Health, Health Science Center, Federal University of Espírito Santo, Vitória, Espírito Santo, Brazil, ufes.br; ^3^ Cassiano Antonio Moraes University Hospital, Federal University of Espírito Santo, Vitória, Espírito Santo, Brazil, ufes.br; ^4^ Department of Public Health, Health Science Center, Federal University of Espírito Santo, Vitória, Espírito Santo, Brazil, ufes.br

**Keywords:** anthropometry, body composition, cancer, muscle mass, sarcopenia

## Abstract

**Background:**

The loss of muscle mass, which is the main component of sarcopenia, is a common condition in women with breast cancer. Thus, novel, simple, accessible methods capable of diagnosing this condition in clinical practice have been investigated.

**Objective:**

To assess the diagnostic accuracy of an anthropometric equation in predicting the skeletal muscle mass index in women with breast cancer.

**Methods:**

An observational cross‐sectional study with women ≥ 20 years of age with up to 12 months since the diagnosis of breast cancer having been submitted to dual‐energy X‐ray absorptiometry (DXA) for the determination of skeletal muscle mass (SMM). Sociodemographic, clinical, and behavioral data were collected. Body mass (kg), height (m), and calf circumference (CC) (cm) were measured. SMM was also obtained through an anthropometric equation. Agreement analysis was performed. Receiver operating characteristic curve analysis, sensitivity, and specificity were calculated.

**Results:**

A total of 135 women were analyzed (55 ± 11.3 years). The sample was predominantly nonwhite (69.7%), with ≤ 6 months since diagnosis (81.5%), insufficiently active (59.3%), and with invasive breast carcinoma (70.4%). The Bland–Altman test showed satisfactory agreement between SMM obtained by the anthropometric equation and the reference method (DXA) for adults and older women. For adults, Cronbach’s alpha was 0.79, the ICC was 0.79, and the mean error was 0.007 kg/m^2^ (95% CI: −0.67 to 0.63). For older women, Cronbach’s alpha was 0.80, the ICC was 0.78, and the mean error was 0.367 kg/m^2^ (95% CI: −1.36–2.10).

**Conclusion:**

Satisfactory agreement was found between SMM obtained by the anthropometric equation and the reference method in both adults and older women. This equation proved to be an effective, simple, and safe tool for the identification of SMM and, consequently, for the screening of sarcopenia in women with breast cancer in clinical practice.

## 1. Introduction

According to the World Health Organization, breast cancer (BC) is the most common form of cancer [1]. The epidemiology of BC reveals the impact of the disease. In addition to being the form of cancer with the highest mortality rate, with 46.8 deaths per 100,000 inhabitants, it is estimated that 847,401 new cases will occur in the world by 2045 [2].

Depending on the tumor type, staging, nutritional factors, and antineoplastic treatments used (surgery, chemotherapy, radiotherapy, and hormone therapy), the pathophysiology of BC leads to significant changes in body composition [3, 4], such as a decline in skeletal muscle mass (SMM), with the consequent development of sarcopenia in this population [5, 6]. Low SMM is a risk factor associated with postoperative complications, a lower response to treatment, and higher mortality rate [5, 7]. According to one study, however, the effect of SMM and density on long‐term survival outcomes in patients with BC remains uncertain [8].

SMM comprises approximately 40% of total body mass and contains between 50% and 75% of body proteins. Its maintenance depends on the balance between protein synthesis and degradation rates [9]. In terms of functions, SMM is responsible for converting chemical energy into mechanical energy to generate force and movement. SMM also contributes to basal energy metabolism by storing amino acids and carbohydrates, to the thermoregulation of the body, as well as oxygen and substrate consumption during physical activity [10], which makes its assessment indispensable in cancer patients.

The different methods for estimating SMM include dual‐energy X‐ray absorptiometry (DXA), computed tomography (CT), bioelectrical impedance analysis (BIA), and magnetic resonance imaging (MRI). However, these methods are expensive and time‐consuming and are not readily accessible in clinical practice [11]. To circumvent this drawback, calf circumference (CC) has been positively associated with SMM in different populations [12–14] and is indicated by the European Working Group on Sarcopenia in Older People (EWGSOP2) as an option for assessing the risk of sarcopenia [15]. CC is an anthropometric measure that is easily applicable to different population groups, genders, and age groups and has been used in various population studies [16–18].

The inclusion of CC in predictive anthropometric equations, such as the one proposed by Santos et al. [19], to estimate SMM can extend and facilitate the identification of low SMM in clinical practice. Despite the relevance of SMM for patients with cancer, there are limitations regarding its identification, due in part to the scarcity of reference or gold standard methods that are accessible in clinical practice. Thus, the investigation of novel, sensitive, effective, low‐cost, easily applicable methods can enable the early screening of low SMM in this population.

### 1.1. Objective

The objective was to analyze the level of agreement of an anthropometric equation in predicting the SMM index in women with BC compared to a gold standard measure.

## 2. Methods

### 2.1. Study Design and Population

An observational cross‐sectional study with convenience sampling was developed from March 2021 to June 2024 at a mastology outpatient clinic of a public hospital located in the city of Vitória, which is the capital of the state of Espírito Santo in southeastern Brazil. This study included women with a minimum age of 20 years and up to 12 months since the diagnosis of BC who did not have metastasis or recurrences and who had been submitted to DXA for the determination of SMM.

### 2.2. Sample Size Calculation

The sample size was calculated with the aid of the G∗Power software, considering an alpha of 5%, a minimum power of 95%, and an effect size of 0.5 for the correlation test, resulting in a minimum sample of 111 participants, to which 20% was added to compensate for possible losses or refusals, totaling a minimum of 133 participants.

A total of 249 women were considered potentially eligible to participate in the study. Among those with whom telephone contact was successful, 188 agreed to participate and were screened for eligibility. Of these, 19 did not meet the inclusion criteria, totaling 169 women assessed. However, 34 did not undergo the body composition assessment by DXA, resulting in a final sample of 135 volunteers.

### 2.3. Data Collection

The participants answered a questionnaire addressing sociodemographic information, lifestyle habits, and clinical history. The sociodemographic variables of interest were age (years), self‐declared skin color (“white,” “black,” “brown,” or “yellow”) [20], marital status (“marital life” and “no marital life”), and schooling (“< 4 years,” “four ≥ 8 years,” “> eight‐ ≤ 11 years” and “> 11 years”) [21].

The lifestyle habits considered were level of physical activity, alcohol consumption, and smoking. Level of physical activity was estimated using the short version of the International Physical Activity Questionnaire (IPAQ) for the Brazilian population [22] and categorized as “sufficient” when the participant performed between 150 and 300 min per week of moderate physical activity or between 75 and 150 min of intense physical activity and “insufficient” when these criteria were not met [23]. Alcohol consumption was self‐reported, and the participants were categorized as “nondrinker,” “former drinker,” or “regularly consumer” [21]. Tobacco use was self‐reported and the participants were categorized as “nonsmoker,” “former smoker,” and “current smoker” regardless of the amount and frequency of use, according to the Consensus on the Approach and Treatment of Smokers [24].

The clinical variables of interest were menopausal status, type of treatment, time since diagnosis, histological subtype, molecular subtype, and clinical staging. Menopause status was defined as “premenopausal” and “postmenopausal.” Type of treatment was categorized as “no treatment” and “with treatment” (surgical, chemotherapy, and radiotherapy). The time since diagnosis was categorized as “≤ 6 months” and “> 6 ≤ 12” months. Histological subtype was categorized as “invasive breast carcinoma,” “invasive ductal carcinoma,” “ductal carcinoma,” and “special subtypes” [21, 25]. Molecular subtype was classified as “luminal A,” “luminal B,” “HER2+,” and “triple negative,” and tumor staging was classified as “O,” “IA or IB,” “IIA or IIB,” or “IIIA, IIIB, or IIIC” [25, 26].

### 2.4. Anthropometric Variables and Body Composition

The anthropometric variables measured were height (m), body mass (kg), waist circumference (WC) (cm), and CC (cm). For the measurement of CC, a nonelastic measuring tape was positioned horizontally around the calf at its maximum circumference, with the individual standing and the legs spaced 20 cm apart, according to the protocol [27]. The body mass index (BMI) (kg/m^2^) was calculated by the ratio of body mass (kg) to height squared (m^2^) and categorized as “underweight,” “normal weight,” “overweight,” “Grade 1 obesity,” “Grade 2 obesity,” or “Grade 3 obesity” [28]. WC < 88 cm was considered adequate, whereas ≥ 88 cm was indicative of abdominal obesity [28]. CC was considered “adequate” if > 33 cm and “low” if ≤ 33 cm [29].

For the analysis of body composition, whole‐body densitometry or DXA was performed to identify the body compartments. The components of interest were total body fat in grams (g) and percentage (%) (BF%), abdominal visceral fat (cm^2^), and appendicular skeletal muscle mass index (ASMMI), which was obtained from the ratio between appendicular skeletal muscle mass (ASMM) (kg) and height squared (m^2^) [15].

The DXA examination was prescheduled with the radiology service of the hospital after a request by the physician in charge and collaborator of this study. DXA was performed using the GE Lunar Prodigy Advance device by a duly trained, certified radiology technician using the GE Encoreà software, Version 14.10, configured to use the reference database of the US National Health and Nutrition Examination Survey (NHANES). The examinations were performed following the manufacturer’s recommendations as well as the recommendations of the Brazilian Society of Clinical Densitometry (SBdens) [30].

The equation proposed by Santos et al. [19] was used to obtain the ASMM from anthropometric measurements, which is composed of age, sex, and CC, as shown below:

ASMM (kg) = −10.427 + [CC (cm) × 0.768] − [age (years) × 0.029] + (female × 0 or male × 7.523) + (white × 0 or black × 2.203 or Mexican American × −0.540 or other × −0.402).

Based on the skin color classification of the Brazilian Institute of Geography and Statistics [20] used in this study, the following correspondences were considered in the application of the equation proposed by Santos et al. [19]: “white” × 0, “black” or “brown” × 2.203, and “yellow” × −0.402.

Low muscle mass was categorized based on the 20th percentile (P20) of the study population itself [31]. Using this cutoff point, muscle mass was considered low when the ASMMI was < 6.23 kg/m^2^ according to DXA and < 6.00 kg/m^2^ according to the anthropometric equation. To facilitate the presentation of the data, the variables were denominated ASMMI 1 when the SMM was obtained by DXA and ASMMI 2 when obtained by the anthropometric equation.

### 2.5. Statistical Analysis

Descriptive analysis was performed. Continuous variables were expressed as mean and standard deviation, whereas categorical variables were expressed as percentage. Bland–Altman plots were used to visualize the level of agreement between the measures, which enabled the assessment of bias, the dispersion of points around the mean, and possible outliers and trends [32, 33]. The intraclass correlation coefficient (ICC) was used to assess the level of agreement between the two measures of the ASMMI. The interpretation of the ICC was based on the Bland–Altman suggestions, with < 0.4 considered unacceptable, 0.41–0.6 denoting good reproducibility, 0.61–0.80 denoting very good reproducibility, and 0.81–1.0 denoting excellent reproducibility [33]. Cronbach’s alpha coefficient was used to measure the reliability and internal consistency of the measures, with a coefficient equal to or greater than 0.70 considered satisfactory [34]. Cohen’s kappa coefficient was calculated to assess the agreement between the prevalence estimates obtained using ASMMI 1 and ASMMI 2. The degree of agreement was interpreted according to the classification proposed by Landis and Koch [35]: poor (< 0.00), slight (0.00–0.19), fair (0.20–0.39), moderate (0.40–0.59), substantial (0.60–0.79), and almost perfect (0.80–1.00) agreement. Using ASMMI 1 as the reference standard, the area under the receiver operating characteristic (ROC) curve (AUC) and its 95% confidence interval (95% CI) were calculated. The diagnostic accuracy of the AUC was interpreted according to the criteria proposed by Metz [36]: poor (0.50–0.80), good (0.80–0.90), and excellent (> 0.90). The data were analyzed using the Statistical Package for the Social Sciences (SPSS) for Windows, Version 22.0, with the significance level set at 5% for all tests.

### 2.6. Ethical Aspects

All individuals participated voluntarily and provided written consent by signing a statement of informed consent in accordance with Resolution 466 of December 8, 2012, of the Brazilian National Board of Health. This study received approval from the Human Research Ethics Committee of the Federal University of Espírito Santo (UFES) in July 2020 (protocol number: 34351120.1.0000.5060; approval certificate number: 4.142.391). The authors confirm that all methods were carried out in accordance with relevant guidelines and regulations (Declaration of Helsinki).

## 3. Results

The sample consisted of 135 participants with a mean age of 55 ± 11.3 years. Adults (64.4%), individuals who lived with a partner (60.7%), those with self‐declared black or brown skin color (66.7%), and those with 4–8 years of schooling (34.8%) predominated in the sample. With regard to lifestyle habits, 59.3% were insufficiently active, 41.5% drank alcohol in the past, and 74.1% had never smoked (Table 1).

**TABLE 1:** Distribution of sociodemographic variables and lifestyle habits of women with breast cancer.

Variables (*n* = 135)	*n* (%)
Age (years)	
Adult (≥ 20–< 60)	87 (64.4)
Older women (≥ 60)	48 (35.6)
Marital status	
With partner	82 (60.7)
Without a partner	53 (39.3)
Color	
White	41 (30.4)
Black/brown	90 (66.7)
Yellow	4 (3.0)
Study time (years)	
< 4	12 (8.9)
4 ≥ 8	47 (34.8)
> 8–≤ 11	44 (32.6)
> 11	32 (23.7)
Physical activity level	
Insufficient	80 (59.3)
Enough	55 (40.7)
Alcohol consumption	
Never drank	50 (37.0)
Former drinker	56 (41.5)
Consumes regularly	29 (21.5)
Smoking habit	
Never smoked	100 (74.1)
Former smoker	28 (20.7)
Smokes regularly	7 (5.2)

In terms of clinical variables, Stage II (44.4%), histological subtype invasive breast carcinoma (70.4%), and luminal molecular subtype B (50.0%) were the predominant findings. Most women had received the diagnosis less than 6 months earlier (81.5%), had already started treatment (56.1%), and were postmenopausal (68.1%) (Table 2).

**TABLE 2:** Characterization of the clinical variables of women with breast cancer.

Variables (*n* = 135)	*n* (%)
Clinical stage^a^	
0	14 (11.1)
AI and IB	32 (25.4)
IIA and IIB	56 (44.4)
IIIA, IIIB, and IIIC	24 (19.0)
Histological subtype	
Invasive breast carcinoma	95 (70.4)
Invasive ductal carcinoma	10 (7.4)
Ductal carcinoma in situ	14 (10.4)
Special subtypes	16 (11.9)
Molecular subtype^b^	
Luminal A	37 (30.8)
Luminal B	60 (50.0)
HER2+	11 (9.2)
Triple negative	12 (10.0)
Diagnostic time (months)	
≤ 6	110 (81.5)
> 6–≤ 12	25 (18.5)
Treatment^c^	
No treatment	58 (43.9)
With treatment	74 (56.1)
Menopausal status	
Premenopausal	43 (31.9)
Postmenopausal	92 (68.1)

^a^ *n* = 126.

^b^ *n* = 120.

^c^ *n* = 132.

The analysis of nutritional status and body composition revealed that 40.0% had some degree of obesity based on the BMI and 82.2% had an adequate CC. Most had a high WC (68.9%), BF% (85.8%), and abdominal visceral fat (53.8%). A total of 20.0% and 18.5% had a low ASMMI according to DXA and the anthropometric equation, respectively (Table 3).

**TABLE 3:** Distribution of variables of nutritional status, body composition, and muscle mass of women with breast cancer.

Variables (*n* = 135)	*n* (%)
BMI	
Low weight	4 (3.0)
Eutrophy	34 (25.2)
Overweight	43 (31.9)
Obesity Grade 1	39 (28.9)
Obesity Grade 2	10 (7.4)
Obesity Grade 3	5 (3.7)
CC	
Adequate	111 (82.2)
Reduced	24 (17.8)
WC	
Normal	42 (31.1)
Increased	93 (68.9)
%BF^a^	
Adequate	19 (14.2)
Elevated	115 (85.8)
Abdominal visceral fat^b^	
Adequate	61 (46.2)
Elevated	71 (53.8)
ASMMI 1	
Adequate	108 (80.0)
Reduced	27 (20.0)
ASMMI 2	
Adequate	110 (81.5)
Reduced	25 (18.5)

*Note:* %BF: body fat percentage.

Abbreviations: ASMMI, appendicular skeletal muscle mass index; BMI, body mass index; CC, calf circumference; WC, waist circumference.

^a^ *n* = 134.

^b^ *n* = 132.

The mean ASMMI among adults was 7.19 ± 1.3 kg/m^2^ using the anthropometric equation and 7.19 ± 1.1 kg/m^2^ using DXA, with an ICC of 0.79 (*p* < 0.001) and a Cronbach’s alpha of 0.79. Among older women, the mean ASMMI was 6.76 ± 1.3 kg/m^2^ using the anthropometric equation and 7.12 ± 1.1 kg/m^2^ using DXA, with an ICC of 0.78 (*p* < 0.001) and a Cronbach’s alpha of 0.80 (Table 4).

**TABLE 4:** Agreement between the appendicular skeletal muscle mass index obtained by the anthropometric equation and the densitometry test obtained by women with breast cancer.

Variables (*n* = 135)	Mean	SD	ICC	*p* value	Cronbach’s alpha
Age					
Adult (kg/m^2^)			0.79	< 0.001	0.79
ASMMI 1	7.19	1.1			
ASMMI 2	7.19	1.3			
Older women (kg/m^2^)			0.78	< 0.001	0.80
ASMMI 1	7.12	1.1			
ASMMI 2	6.76	1.3			
Clinical stage^a^					
0			0.658	0.032	0.658
ASMMI 1	7.31	1.10			
ASMMI 2	7.14	1.28			
AI and IB			0.779	< 0.001	0.779
ASMMI 1	7.29	0.92			
ASMMI 2	7.06	1.08			
IIA and IIB			0.843	< 0.001	0.843
ASMMI 1	7.16	1.18			
ASMMI 2	7.05	1.37			
IIIA, IIIB, and IIIC			0.764	0.001	0.764
ASMMI 1	7.00	1.20			
ASMMI 2	6.98	1.56			
Histological subtype					
Invasive breast carcinoma			0.792	< 0.001	0.792
ASMMI 1	7.11	1.15			
ASMMI 2	7.00	1.27			
Invasive ductal carcinoma			0.737	0.030	0.737
ASMMI 1	7.10	0.68			
ASMMI 2	6.75	0.83			
Ductal carcinoma in situ			0.812	0.002	0.812
ASMMI 1	7.60	1.01			
ASMMI 2	7.25	0.99			
Special subtypes			0.786	0.002	0.786
ASMMI 1	7.21	1.06			
ASMMI 2	7.40	1.77			
Molecular subtype^b^					
Luminal A			0.856	< 0.001	0.856
ASMMI 1	7.22	1.26			
ASMMI 2	6.82	1.20			
Luminal B			0.810	< 0.001	0.810
ASMMI 1	7.04	1.04			
ASMMI 2	7.08	1.31			
HER2+			0.750	0.020	0.750
ASMMI 1	7.66	1.02			
ASMMI 2	7.41	1.10			
Triple negative			0.823	0.004	0.823
ASMMI 1	7.23	1.15			
ASMMI 2	7.01	1.48			
Diagnostic time (months)					
≤ 6			0.807	< 0.001	0.807
ASMMI 1	7.13	1.11			
ASMMI 2	6.96	1.29			
> 6–≤ 12			0.684	0.003	0.684
ASMMI 1	7.34	1.03			
ASMMI 2	7.38	1.23			
Treatment^c^					
No treatment			0.848	< 0.001	0.848
ASMMI 1	7.12	1.22			
ASMMI 2	6.96	1.36			
With treatment			0.725	< 0.001	0.725
ASMMI 1	7.20	0.99			
ASMMI 2	7.10	1.24			
Menopausal status					
Premenopausal			0.787	< 0.001	0.787
ASMMI 1	7.37	1.02			
ASMMI 2	7.22	1.08			
Postmenopausal			0.787	< 0.001	0.787
ASMMI 1	7.08	1.12			
ASMMI 2	6.95	1.36			
BMI					
Underweight/normal weight			0.646	< 0.001	0.646
ASMMI 1	6.31	0.71			
ASMMI 2	5.87	0.82			
Overweight/obesity			0.651	< 0.001	0.651
ASMMI 1	7.57	0.94			
ASMMI 2	7.48	1.14			

Abbreviations: ASMMI, appendicular skeletal muscle mass index; BMI, body mass index; ICC, intraclass correlation coefficient; SD, standard deviation.

^a^ *n* = 126.

^b^ *n* = 120.

^c^ *n* = 132.

The agreement analysis between the ASMMI obtained by the anthropometric equation and by DXA, stratified by clinical variables and BMI, demonstrated agreement between the methods, regardless of clinical factors and BMI category. The ICC ranged from very good to excellent reproducibility across the subgroups. Cronbach’s alpha values were consistent with the ICC results, confirming good internal consistency between the methods (Table 4).

**FIGURE 1:**
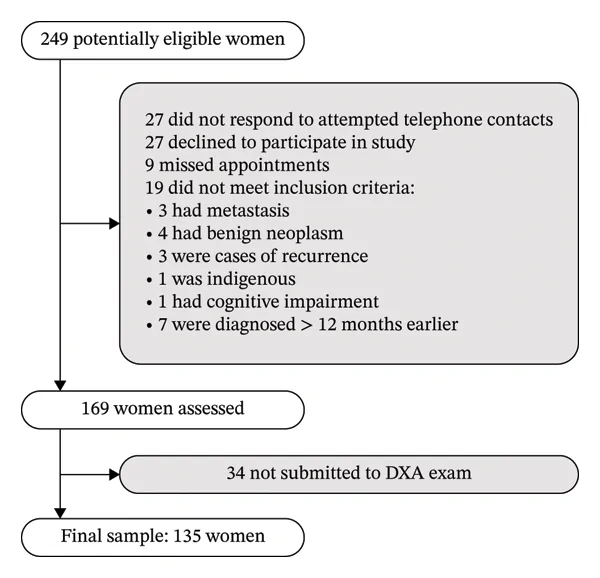
Flowchart of participant selection.

**FIGURE 2:**
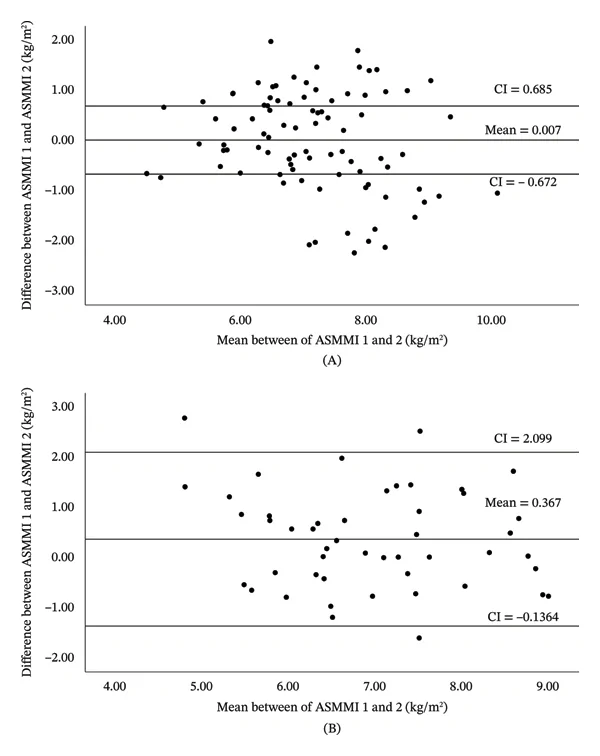
Bland–Altman limits of agreement between appendicular skeletal muscle mass index (ASMMI) (Kg/m^2^) in adults (A) and older women (B) with breast cancer. Legend: ASMMI: appendicular skeletal muscle mass index; CI: confidence interval.

Agreement between methods was expressed graphically in Bland–Altman plots considering adults and older women. The mean error between measurements (estimated bias) was 0.007 kg/m^2^ (95% CI: −0.67 to 0.63) among the adults (Figure 1) and 0.367 kg/m^2^ (95% CI: −1.36–2.10) among the older women (Figure 2).

**FIGURE 3:**
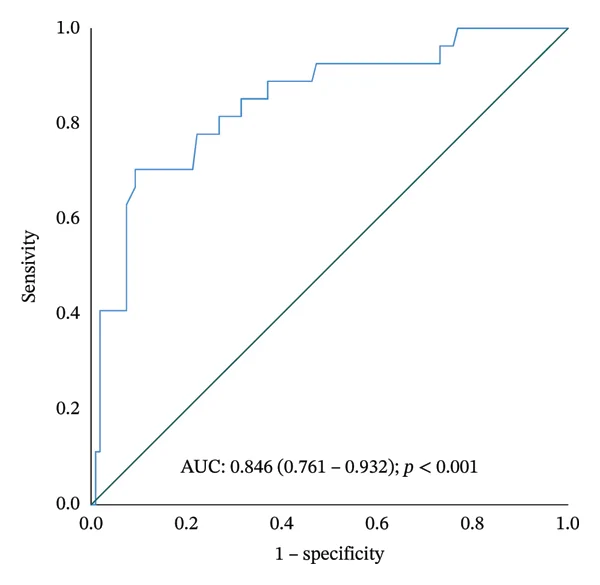
Receiver operating characteristic (ROC) curve comparing the appendicular skeletal muscle mass index obtained by the anthropometric equation with the appendicular skeletal muscle mass index obtained by reference methods.

The diagnostic performance of ASMMI 2 was further evaluated using ROC curve analysis. The AUC was 0.846 (95% CI: 0.761–0.932; *p* < 0.001), with a sensitivity of 70% and a specificity of 90% (Figure 3).

In supporting analyses, the agreement between ASMMI 1 and ASMMI 2 was assessed in the overall sample and after stratification by BMI into the underweight/normal‐weight and overweight/obesity categories. Moderate agreement between the two methods was observed in all analyses (Supporting Tables 1 and 2).

## 4. Discussion

The present study analyzed a population of women with BC composed predominantly of adults and overweight individuals, as commonly observed in this group [37]. The agreement analysis revealed that the anthropometric equation based on CC proposed by Santos et al. [19] demonstrated good to excellent agreement and reproducibility for obtaining SMM compared to the reference method (DXA) both for adults and for older women and confirmed by AUC analysis. The use of alternative methods to identify low SMM, which is the main component of sarcopenia, can expand and facilitate the screening of this condition in women with BC.

Among the reference methods for identifying SMM, DXA is a noninvasive instrument that quantifies muscle mass and adjusts SMM to body size using height squared [15]. CT is the gold standard method for assessing body composition on the tissue and organ level but is contraindicated for repeated assessments due to exposure to high doses of ionizing radiation [38]. Like CT, MRI is a gold standard method but is not commonly used for the determination of body composition due to the high cost, lack of portability, and need for highly trained personnel, in addition to not having well‐defined cutoff points for low SMM [39]. BIA estimates body compartments by resistance to the flow of a low‐amplitude electric current and is relatively accessible but depends on specific predictive equations for the population studied [38].

Anthropometric variables have been investigated as an alternative method for assessing body composition. With regard to the identification of SMM, different measures have been studied as markers of SMM in diverse populations, such as arm muscle area [40], adductor pollicis muscle thickness [41, 42], arm circumference [43–46], and the CC [12, 15–17, 29, 47], which remains one of the most prominent anthropometric variables studied.

The use of simple measures, that is, those that can be performed by any professional trained in anthropometric assessment, such as equations involving CC [19], contributes to the identification of low SMM in clinical practice by combining low cost and easy applicability in contrast to expensive, inaccessible methods, such as DXA, CT, MRI, and BIA [48, 49]. Moreover, CC is recommended by the EWGSOP2 [15] as a screening tool for sarcopenia. According to Barbosa‐Silva et al. [50], low CC is a good indicator of muscle loss, low functional capacity, and the risk of frailty, considering the substantial volume of skeletal muscle located in the lower limbs.

Other studies have associated CC with SMM in different populations. Grili et al. [12] analyzed postmenopausal women and found a positive correlation between these two variables. Kawakami et al. [51] sought to validate an anthropometric equation to estimate ASMM based on body composition and found a strong positive correlation between CC and SMM, as measured by DXA, in Japanese men and women 40 years of age or older. Analyzing older people living in a Finnish community, Kerminen et al. [13] measured SMM using DXA as the gold standard and compared the performance of CC, SARC‐F, SARC‐CalF, and CC adjusted by BMI for detecting cases of sarcopenia. The authors found that CC performed better than the other tools analyzed.

Lee et al. [11], in a study involving healthy adults from the United States and Canada, proposed a predictive anthropometric equation incorporating CC as an indicator of SMM to estimate total SMM and reported high sensitivity and specificity. In subsequent studies, Felipe et al. [52] found that this predictive equation demonstrated the expected agreement in estimating SMM in postmenopausal women, and Santos et al. [25] showed that the equation presented sufficient diagnostic capacity to be used as an alternative for identifying sarcopenia in women with BC. Therefore, these findings underscore the need to further investigate other anthropometric equations that incorporate this measure.

The equation proposed by Santos et al. [19] used data from 1999 to 2006 from more than fifteen thousand adults of the US NHANES, which was a cross‐sectional study with a representative sample that investigated the health and nutrition status of American children and adults. Based on different proposals and validation analyses, the equation consisting only of CC, sex, race, and age as independent variables was the one that obtained the best performance in the estimation of SMM among the models studied by the authors. The study showed that the anthropometric equation achieved high agreement and was able to explain nearly 90% of the variation in the SMM measured by DXA. However, despite the large sample, the analyses do not ensure the representativeness of the results in terms of the distribution of sex, age, and ethnicity or reference values for all populations. The present investigation is the first to evaluate the anthropometric equation proposed by Santos et al. [19] for estimating SMM in women with BC. The results demonstrated satisfactory diagnostic performance and suggest that this method may represent a practical alternative for large‐scale screening of low SMM.

The assessment of SMM in the context of cancer is essential, as these patients are particularly at risk of weakness and significant muscle loss due to low nutrient intake, metabolic abnormalities associated with the disease [53], antineoplastic treatments, and the need for hospitalization [54]. Several studies have highlighted the association between cancer and the risk of muscle wasting and sarcopenia [54].

Systematic review and meta‐analysis studies have reported a positive association between SMM and clinical outcomes. Trejo‐Avila et al. [55] demonstrated that low muscle mass or strength was predictive of lower quality of life and higher rates of pre‐ or postoperative complications and morbidities in patients with colorectal cancer. Beaudart et al. [54] analyzed studies that investigated potential associations between muscle mass/strength and healthcare costs in patients with different types of cancer, and identifying higher costs and higher risks of hospital readmissions among patients with lower muscle mass.

Analyzing 9863 patients with BC, Jang et al. [56] found that low muscle mass was associated with an increased risk of side effects related to treatment and the modification of recommended treatment plans in patients with BC. On the other hand, Deng, Wang, and Li [8] analyzed 13 studies of patients with BC with and without metastasis and found an uncertain effect of SMM and skeletal muscle density on long‐term survival. However, the studies analyzed were not of high quality, suggesting the need for prospective cohort studies.

Despite the relevance of low muscle mass in this population, gaps and limitations are found in the investigation. The difficulty in gaining access to gold standard methods in clinical practice can significantly contribute to the underdiagnosis of this condition, in addition to compromising the effectiveness of clinical screening. Thus, the adoption of simple, easily accessible, noninvasive methods, such as the measurement of CC, a low value of which has been associated with the loss of muscle mass, can be useful in identifying SMM. The use of CC to identify low SMM can assist in screening for sarcopenia in this population, especially in clinical settings with limited resources, contributing to more timely and effective interventions.

### 4.1. Limitations

The fact that the equation is not validated for the Brazilian population is a limitation of this study. However, this is the first study to use the anthropometric equation proposed by Santos et al. [19] to determine SMM in women with BC. The results confirm the accuracy of the equation. Another limitation is the study design, which does not enable extrapolating the results to the general population of women with BC, as the study was carried out with a group of women from a single outpatient clinic belonging to the secondary service of the public health system.

### 4.2. Strengths

The use of CC with the anthropometric equation proposed by Santos et al. [19] is a promising, simple, fast, low‐cost, noninvasive method for identifying low SMM, which may be useful for screening for sarcopenia, in addition to being a measure recommended by the European Consensus [15]. Moreover, Santos et al. [19] considered both the adult and older adult populations, which differentiates the equation from that proposed by Lee et al. [11], who only considered only the older population.

## 5. Conclusion

The anthropometric equation analyzed achieved satisfactory agreement and diagnostic accuracy with the reference method for estimating skeletal muscle mass in adults and older women with BC. These results demonstrate the clinical potential of this tool as a viable, easily implemented option for screening for sarcopenia in women with BC. The adoption of simple but accurate methods can facilitate the early identification of muscle loss, thus enabling more timely nutritional and functional interventions, with a positive impact on the prognosis and quality of life of these patients.

## Funding

No funding was received for this manuscript.

## Conflicts of Interest

The authors declare no conflicts of interest.

## Supporting Information

Additional supporting information can be found online in the Supporting Information section.

## Supporting information


**Supporting Information** Table S1. Concordance between ASMMI 1 and ASMMI 2 in women with breast cancer (*n* = 135). Table S2. Concordance between ASMMI 1 and ASMMI 2 in women with breast cancer according to BMI category (*n* = 135).

## Data Availability

The data that support the findings of this study are available from the corresponding author upon reasonable request.
