# Supplementary material for: Skeletal Muscle Mass in Women With Breast Cancer: Evaluation of Agreement Between Different Methods
Source: J Nutr Metab. 2026 Aug 24;2026:6646504. doi: 10.1155/jnme/6646504 (PMC13501393; doi:10.1155/jnme/6646504)
Supplement: Supplementary file 1 [file JNME-2026-6646504-s001.docx]

**Table S1.** Concordance between ASMMI 1 e ASMMI 2 in women with breast cancer (n=135).

|  | ASMMI 1 | |  |  |
| --- | --- | --- | --- | --- |
| ASMMI 2 | Adequate | Reduced | Kappa | p value |
| Adequate | 100 (90.9) | 10 (9.1) | 0.57 | **0.009** |
| Reduced | 8 (32.0) | 17 (68.0) |  |  |

ASMMI: Appendicular Skeletal Muscle Mass Index

**Table S2.** Concordance between ASMMI 1 e ASMMI 2 in women with breast cancer acoording BMI category (n=135).

| Low weight/ Eutrophy | | | | |
| --- | --- | --- | --- | --- |
|  | ASMMI 1 | |  |  |
| ASMMI 2 | Adequate | Reduced | Kappa | p value |
| Adequate | 13 (68.4) | 6 (31.6) | 0.42 | **0.009** |
| Reduced | 5 (26.3) | 14 (73.7) |  |  |
| Overweight/Obesity | | | | |
|  | ASMMI 1 | |  |  |
| ASMMI 2 | Adequate | Reduced | Kappa | p value |
| Adequate | 87 (95.6) | 4 (4.4) | 0.42 | **< 0,001** |
| Reduced | 3 (50.0) | 3 (50.0) |  |  |

ASMMI: Appendicular Skeletal Muscle Mass Index.
